# Allopreening in birds is associated with parental cooperation over offspring care and stable pair bonds across years

**DOI:** 10.1093/beheco/arx078

**Published:** 2017-06-09

**Authors:** Elspeth Kenny, Tim R Birkhead, Jonathan P Green

**Affiliations:** a Department of Animal and Plant Sciences, Alfred Denny Building, University of Sheffield, Western Bank, Sheffield, S10 2TN, UK, and; b The Edward Grey Institute of Field Ornithology, Department of Zoology, University of Oxford, The John Krebs Field Station, Wytham, Oxford, OX2 8QJ, UK

**Keywords:** allopreen, divorce, offspring care, pair bond, parental cooperation

## Abstract

Individuals of many species form bonds with their breeding partners, yet the mechanisms maintaining these bonds are poorly understood. In birds, allopreening is a conspicuous feature of interactions between breeding partners and has been hypothesized to play a role in strengthening and maintaining pair bonds within and across breeding attempts. Many avian species, however, do not allopreen and the relationship between allopreening and pair bonding across species remains unexplored. In a comparative analysis of allopreening and pair bond behavior, we found that allopreening between breeding partners was more common among species where parents cooperate to rear offspring. The occurrence of allopreening was also associated with an increased likelihood that partners would remain together over successive breeding seasons. However, there was no strong evidence for an association between allopreening and sexual fidelity within seasons or time spent together outside the breeding season. Allopreening between partners was also no more common in colonial or cooperatively breeding species than in solitary species. Analyses of evolutionary transitions indicated that allopreening evolved from an ancestral state of either high parental cooperation or high partner retention, and we discuss possible explanations for this. Overall, our results are consistent with an important role for allopreening in the maintenance of avian pair bonds.

## Lay Summary

Preening one’s partner is important for staying together and sharing offspring care. Grooming or preening a partner is widespread in primates and birds. We tested the idea that this behavior (allopreening) in birds plays a role in maintaining partner relationships. Bird species that allopreen were more likely to share parental duties and more likely to breed with the same partner in consecutive breeding seasons, compared to species that do not allopreen.

Twitter: @ElspethKenny

## INTRODUCTION

Types of social relationship between males and females vary from promiscuous species with no bond to long-term social monogamy, yet behaviors associated with these different types of relationship remain poorly understood. In birds, one behavior that may play an important role in maintaining a social relationship between partners is allopreening (mutual preening), whereby the bill is used to preen the partner’s feathers. In primates, the analogous behavior, allogrooming, is exchanged reciprocally between group members or traded for other commodities which strengthens relationships (Seyfarth and Cheney 1984; Henzi and Barrett 1999; Tiddi et al. 2012) and ultimately increases participant fitness (Dunbar 1991; Silk et al. 2009; Silk et al. 2010; McFarland and Majolo 2013) by reducing stress and removing ectoparasites (Boccia et al. 1989; Tanaka and Takefushi 1993; Aureli et al. 1999; Wittig et al. 2008). In contrast, the social function of allopreening in birds is considerably less well understood. This is particularly surprising given the striking variation across bird species in the occurrence of allopreening: in some species, allopreening is a highly conspicuous feature of breeding partner interactions; in others this behavior is entirely absent.

Allopreening can aid ectoparasite removal (Brooke 1985; Villa et al. 2016). However, if hygiene is the primary function of allopreening, why does this behavior occur in certain species only? One possibility is that gregarious species, in which frequent physical contact among individuals facilitates parasite transmission, are more prone to ectoparasites (Boyd 1951). Alternatively, the removal of ectoparasites by allopreening may provide long-term fitness benefits by maintaining the health of both breeding partners in species with long-term pair bonds (Black 1996).

A third explanation for the uneven distribution of allopreening across species is that allopreening serves a different social function. An early review by Harrison (1965) argued that allopreening strengthens the bond between breeding partners, but examined only a small number of species and did not determine the statistical association between pair bond strength and allopreening across species. The notion that allopreening reinforces pair bonds has gained widespread acceptance (e.g., Harrison and Harrison 1997; Dagg 2011; Mandal 2015), but to date this hypothesis has been examined in only a handful of species. In buff-breasted wrens *Cantorchilus leucotis* and cockatiels *Nymphicus hollandicus*, allopreening is associated with partner retention across breeding seasons and coordination over incubation respectively (Spoon et al. 2006; Gill 2012), but in a third species, the common guillemot *Uria aalge*, allopreening appears to play no role in pair bond maintenance (Lewis et al. 2007). Outside the pair bond, evidence for a social function of allopreening comes from studies of cooperatively breeding green woodhoopoes *Phoeniculus purpueus*, which have been reported to increase allopreening among group members following territorial conflicts with neighboring groups (Radford 2008; Radford 2011). However, the lack of detailed observations for most species denies us a broad understanding of variation in allopreening across birds, and in particular, how it might influence the establishment and reinforcement of social relationships between breeding partners.

Here, we carry out a comparative analysis of allopreening within breeding bird pairs, testing the hypothesis that across species the presence of allopreening is associated with strong pair bonds. Specifically, we explored the association between allopreening and 4 measures of pair bond strength: 1) parental cooperation in offspring care duties; 2) retention of breeding partners in consecutive breeding attempts; 3) sexual fidelity within seasons, and 4) time spent together outside the breeding season.

## METHODS

### Data collection

We searched published sources for information on the following aspects of avian pair bonds: parental cooperation over offspring care (using scores from Remeš et al. 2015), duration of offspring care (days until independence), annual divorce rate (number of divorced pairs divided by the total number of pairs where both partners survived from one year to the next), extra-pair paternity (EPP; percentage of broods containing extra-pair offspring) and duration of the pair bond throughout the year (continuous or part-time relationship). We also recorded whether species typically exhibited solitary breeding or colonial or cooperative breeding, to test whether allopreening was more common between pairs breeding in groups, as has been suggested by previous authors (Harrison 1965; Spottiswoode 2008). For those species where information on one or more of the above variables was available, we then searched the literature to establish the presence or absence of allopreening within breeding pairs. We used this binary measure of allopreening because rates of allopreening are available for a few species only. We implemented extensive Web of Science and Google Scholar searches using the terms “allo*preen*”, “mutual preen*”, “allo*groom*”, and “mutual groom*”, in combination with the species’ binomial nomenclature and common name(s). We also used information sources at the Alexander Library of Ornithology (Bodleian Libraries, University of Oxford, UK), and contacted researchers involved in long-term studies of particular species where insufficient published data existed. We gathered information on the occurrence of allopreening in a total of 503 species from 116 families. For full details of data collection and sources for all variables see electronic Supplementary Text S1 and Supplementary Table S1. Differences in sample sizes between analyses reflect differences in the availability of estimates for variables among species.

### Phylogenetic analyses

Rather than basing our analyses on a single phylogenetic tree and assuming this tree was known without error, we instead used a distribution of 100 phylogenetic trees extracted from www.birdtree.org (Hackett constraint, Jetz et al. 2012, Phylogenetic Trees supp.). We used the Markov chain Monte Carlo (MCMC) (Hadfield and Nakagawa 2010) approach implemented in the “mulTree” package (Guillerme and Healy 2014) in R (v. 3.2.2, R Core Team 2013), which runs the models on all 100 trees and summarises the resulting 100 parameter estimates. We estimated the phylogenetic signal by optimizing the λ parameter in a phylogenetic generalized least-squares (PGLS) approach (Pagel 1999; Orme et al. 2013). We ran separate MCMC models to test the relationship between allopreening (present/absent) and each measure of pair bond strength: parental cooperation score, duration of offspring care, divorce rate (with mortality rate as a covariate), extra-pair paternity, partnership duration (continuous or part-time), colonial breeding (yes/no) and cooperative breeding (yes/no). In addition, to confirm the effects obtained from the separate models, we also ran a full model containing all predictors on a subset of 37 species for which information on all variables was available. Parameter estimates were considered statistically significant when 95% confidence intervals did not include 0.

### Evolutionary processes

Based on the results of the above analyses, we tested for correlated evolution between allopreening and (a) divorce and (b) parental cooperation using the BAYESTRAITS DISCRETE module with MCMC sampling (Pagel 1994). As BAYESTRAITS requires binary characters, we assigned species that were equal to or greater than the median level of divorce as “high divorce rate” and those that were less as “low divorce rate”, and likewise for parental cooperation scores (for a similar approach, see e.g. Cornwallis et al. [2010] and Downing et al. [2015]). To check the sensitivity of this categorization we repeated the analyses with species divided by 10% above and below the median (see electronic Supplementary Text S1). Transition rates were assessed by running a Reverse Jump model (Pagel and Meade 2006), and we accounted for phylogenetic uncertainty by including the same 100 trees used in the above analyses. We compared model support using Bayes factors estimated from a stepping stone sampling procedure (Xie et al. 2011). Full details are available in the electronic Supplementary Text S1.

## RESULTS

**Figure 1:**
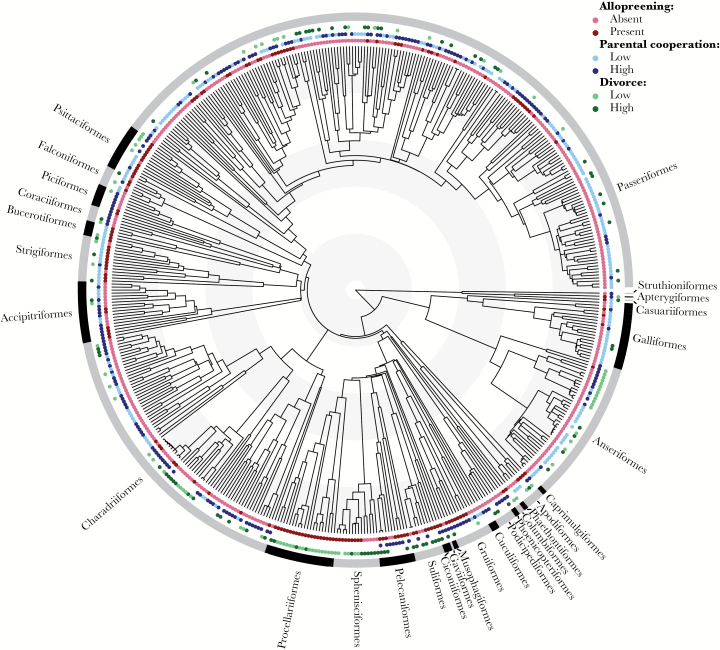
The phylogenetic distribution of allopreening, divorce and parental cooperation in birds (*n* = 503). “High” and “low” divorce and parental cooperation are categorised as higher or lower than the median rate/score.

We found the presence of allopreening to be typically conserved within avian orders but variable between orders, as demonstrated by the strong phylogenetic signal (Pagel’s λ = 0.83; Figure 1). For example, allopreening occurs widely within both the Procellariiformes (albatrosses and petrels) and Psittaciformes (parrots) but is almost entirely absent from the Anseriformes (ducks and geese). In several orders, however, there is substantial variation in allopreening between genera, for example within the Sphenisciformes (penguins) and Accipitriformes (hawks, eagles, and allies).

**Figure 2:**
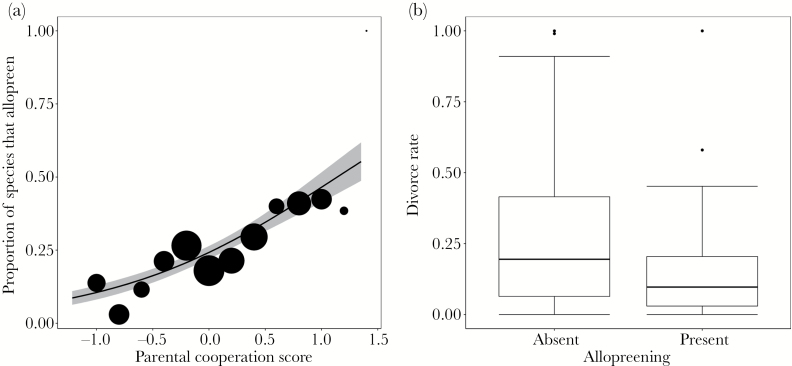
(a) Allopreening is more common among species where parents cooperate over offspring care (data from 106 allopreening and 312 non-allopreening species). Higher parental cooperation scores represent more equal contributions from both pair members to offspring care duties; lower parental cooperation scores indicate unequal contributions from pair members to offspring care duties. Point sizes represent the number of species that were assigned a given parental cooperation score (from Remeš et al. 2015). The grey area shows 95% confidence intervals. (b) Allopreening species have lower divorce rates than non-allopreening species (*n* = 174). Central lines represent median values, the top and bottom lines of the box represent the first and third quartiles and vertical lines represent approximately 2 standard deviations around the interquartile range (circles denote outliers).

Controlling for phylogeny, we found that allopreening was associated with greater cooperation between parents over offspring care (*n* = 418 species, Table 1, Figure 2a). This association remained significant when tested in the full model (*n* = 37 species, electronic Supplementary Table S2). Parental cooperation did not vary with the duration of offspring care (*r* = 0.004, *t* = 0.04, df = 112, *P* = 0.96) and there was no association between allopreening and offspring care duration (posterior mode = 0.004, 95% CIs = −0.009 to 0.02, n = 184).

**Table 1:** Allopreening is significantly associated with parental cooperation in 418 avian species. (Estimates are modal estimates from 100 models. Lower CI = lower 95% confidence interval. Upper CI = upper 95% confidence interval. Parameter estimates were considered statistically significant when 95% confidence intervals did not include 0, denoted by bold typeface. Residual variance was set to 1.)

	Estimate (β)	Lower CI	Upper CI
Fixed terms			
Intercept	–0.78	–3.83	2.33
**Parental cooperation**	**1.92**	**0.90**	**3.05**
Random terms			
**Phylogenetic variance**	**13.74**	**6.89**	**23.62**

We found that divorce rates were significantly lower in allopreening species than in non-allopreening species (median = 9.7% vs. 19.5%, *n* = 174, Table 2, Figure 2b). There was no association between the likelihood of divorce and cooperation score (posterior mode = −0.03, 95% CIs = −0.14 to 0.08, *n* = 92), indicating that allopreening varies independently with both divorce and parental cooperation. Despite mortality being a significant correlate of divorce (Jeschke and Kokko 2008), annual mortality rate did not significantly predict allopreening when included as a covariate in the divorce model (Table 2) indicating that the relationship between allopreening and divorce is not driven by mortality. When analyzing divorce together with other pair bond measures in the full model, the association between allopreening and divorce was no longer significant (*n* = 37, electronic Supplementary Table S2). This was likely due to the reduction in sample size, and hence power, of the full model, rather than the influence of other predictors: when the effect of divorce was analyzed separately on the same subset of species, the relationship with allopreening was again nonsignificant (posterior mode = −0.59, 95% CIs = −6.49 to 4.88, *n* = 37).

**Table 2:** Allopreening is significantly associated with divorce rates in 174 avian species. (Column heads as explained in table 1. Parameter estimates were considered statistically significant when 95% confidence intervals did not include 0, denoted by bold typeface. Residual variance was set to 1.)

	Estimate (β)	Lower CI	Upper CI
Fixed terms			
Intercept	0.18	–3.26	3.69
**Divorce**	**–3.58**	**–7.20**	**–0.35**
Mortality	–0.54	–6.05	5.23
Random terms			
**Phylogenetic variance**	**11.48**	**4.68**	**21.68**

Although allopreening species were more likely to retain partners across breeding seasons, we found no evidence for an association between allopreening and sexual fidelity to social partners within breeding seasons, measured as the rate of EPP (posterior mode = −0.011, 95% CIs = −0.065 to 0.042, *n* = 74). However, when EPP was included as a predictor in the full model, we detected a weak but significant effect, with allopreening species showing higher rates of EPP than non-allopreening species (*n* = 37, electronic Supplementary Table S2). The reason for this discrepancy is unclear. One explanation could be that a relationship between EPP and allopreening does indeed exist, but that it is weak and only evident when accounting for variance in allopreening explained by other variables. Consistent with this, when the effect of EPP was analysed separately on the same subset of species, the relationship with allopreening was nonsignificant (posterior mode = 0.037, 95% CIs = −0.021 to 0.096, *n* = 37). Alternatively, the presence of an effect of EPP in the reduced subset of 37 species but not in the full set of 74 species for which EPP data were available may be spurious and reflect an unidentified bias in the reduced set of species analyzed in the full model.

Breeding partners in many species maintain bonds during the breeding season only, but some species also remain together throughout the non-breeding season. The latter had significantly lower divorce rates (median = 5.60% vs. 23.25%, posterior mode = −9.30, 95% CIs = −14.75 to −4.90, *n* = 137) but were not more likely to allopreen than the former (posterior mode = 1.013, 95% CIs = −0.63 to 1.57, *n* = 137). Finally, we also found no association between allopreening between breeding partners and group sociality. Specifically, allopreening between breeding partners was not more common in colonially (posterior mode = 0.92, 95% CIs = −0.72 to 2.59, *n* = 166) or cooperatively (posterior mode = −0.86, 95% CIs = −2.30 to 0.56, *n* = 358) breeding species than in solitary-breeding species.

In analyses of evolutionary transitions, a model that assumed correlated evolution of allopreening and parental cooperation provided a better fit to the data than a model assuming independent evolution (likelihood = −427.46 vs. −437.07, Bayes factor = 25, df = 4, *P* < 0.001, *n* = 418). In these analyses, estimates of the rates of evolutionary transitions were somewhat dependent upon the method of classifying high and low parental cooperation. When categorizing high and low parental cooperation as higher or lower than the median cooperation score across species, the estimated rate of transition to gain allopreening was close to zero for ancestors with little or no parental cooperation over offspring care, while all other transitions were equally likely (electronic Supplementary Figures S1a and S2a, Supplementary Table S3a). Similar results were obtained when categorizing high and low cooperation as greater or lesser than 10% below the median, but not when categorizing high and low cooperation as greater or lesser than 10% above the median. Nonetheless, among the 70 species that switched between the “low” and “high” parental cooperation categories in these 2 models, most of those with higher parental cooperation scores allopreen and most of those with lower parental cooperation scores do not allopreen.

For partner retention, a model assuming correlated evolution of allopreening and divorce provided a better fit to the data than a model assuming independent evolution (likelihood = −208.97 vs. −213.99, Bayes factor = 10, df = 4, *P* < 0.04, *n* = 174). Transition rates were robust to the method of categorizing high and low divorce rates (Table S3b). The rate of transition to gain allopreening behavior was close to zero for ancestors with high divorce rates and the rate of transition to lose allopreening behavior was close to zero for ancestors with low divorce rates. All other transitions were equally likely (electronic Supplementary Figures S1b and S2b, Supplementary Table S3b). Overall, these results suggest that allopreening evolved either from a state of high parental cooperation or high partner retention.

## DISCUSSION

Controlling for phylogeny, we found that the presence of allopreening was significantly associated with cooperation by parents over offspring care. We also found support for a relationship between allopreening and partner retention across years, with allopreening species exhibiting lower rates of divorce between breeding seasons. Within breeding seasons, there was also some suggestion of an association between allopreening and sexual fidelity, but the apparent effect we observed was weak and inconsistent between analyses, preventing us from drawing clear conclusions. Of course, the results of our comparative analysis are correlative and do not directly address causal links between pair bond strength and allopreening, nor do they rule out the possibility that other, unidentified factors are responsible for driving the observed associations between allopreening and pair bond strength. However, the results of our analyses of evolutionary transitions indicate that allopreening most likely evolved when divorce rates were low or cooperation over offspring care was high, lending support to the idea that allopreening may have evolved as a mechanism to maintain social relationships in species where reproductive success depends upon strong pair bonds between breeding partners.

Parental cooperation was not correlated with divorce rate in our study. This is in contrast with a number of within-species studies that have shown 1) re-mating with the same partner promotes coordination of breeding activities (Handel and Gill 2000; Griggio and Hoi 2011; Sánchez-Macouzet et al. 2014; Leu et al. 2015) and 2) divorce is more common when partners fail to provide adequate parental care (Moody et al. 2005). Although joint parental investment in offspring care might be expected to coevolve with stable pair bonding across breeding attempts, theoretical modelling has shown this will depend on the costs to partners of forming such a bond (Song and Feldman 2013). For example, waiting for a late-arriving partner at the start of a breeding season may result in lost breeding opportunities, while increased disease transmission or competition from resources may disfavor breeding partners remaining in close contact in the non-breeding season. Alternatively, our analyses may have lacked power to detect a relationship between divorce and parental cooperation among the relatively small number of species for which data on both variables were available.

Our analyses indicate that relative contributions to parental care and divorce rates coevolved with allopreening, and that allopreening evolved either from a state of high parental cooperation or low divorce. This finding poses the question: if, as our results suggest, allopreening evolved to strengthen the pair bond, why would allopreening be selected for in species where the pair bond was already strong? One possibility is that where it is adaptive to share offspring care duties or to re-pair with the same partner, it may be adaptive to care for the partner’s health by engaging in preening to remove ectoparasites. Alternatively, allopreening may serve to reinforce pair bonds by facilitating cooperation between partners or long-term recognition. In birds and mammals, the pituitary hormone oxytocin appears to play important roles in both contexts (Williams et al. 1994; Ross and Young 2009; Insel 2010; Klatt and Goodson 2013; Romero et al. 2014), and a number of other hormones, including testosterone (Hirschenhauser 2012), vasopressin (Lim and Young 2006) and endorphins (Keverne et al. 1989; Dunbar 2010), have also been implicated in the development of pair bonds. Although our study is correlative and does not address the underlying mechanisms linking allopreening with pair bond behavior, one possibility is that allopreening between partners stimulates the release of hormones such as oxytocin, which in turn initiates pair bond formation and facilitates learning of breeding partner identity. Consistent with this idea, research on primates has shown that affiliative interactions among close social partners are associated with an increase in levels of peripheral oxytocin (Crockford et al. 2013) and the release of endorphins (Keverne et al. 1989). Studies of primates have also shown that both affiliative contact between individuals and the subsequent increases in levels of oxytocin and endorphins are effective in reducing stress (Boccia et al. 1989; Sapolsky et al. 1997; Aureli et al. 1999; Carter et al. 1999; Taylor 2006; Wittig et al. 2008; Aureli and Yates 2010), which is likely to play an important role in reinforcing pair bonds. Allopreening may serve a similar function in birds, though more research on the physiological changes that occur in response to allopreening and their downstream effects is required to test this idea.

Although we identified significant associations between allopreening and partner retention and parental cooperation, there were exceptions to the general trends: for example, riflemen *Acanthisitta chloris* pairs do not allopreen yet have high mate retention, and greater painted-snipe *Rostratula benghalensis* preen their partners but show uniparental care. Thus, allopreening is neither necessary nor sufficient for either equal parental investment or high mate retention. In the absence of allopreening, other pair behaviors, such as courtship feeding or duets, may have similar or complementary effects on parental care strategies or mate retention (e.g. Lack 1940; Boucaud et al. 2016). Interestingly, we found that allopreening was not more likely among species that maintain pair bonds throughout the non-breeding season than those that come together during the breeding season only. This does not necessarily contradict the idea that allopreening is important in pair bond maintenance, however. Pair bond reinforcement through allopreening and other behaviors may be more important in the breeding season, when the ability to provide effective parental care may depend upon close coordination of breeders’ activities and hence may be compromised by exploitation of one parent by the other (e.g., through brood desertion) (Houston et al. 2005). Another possibility is that allopreening has downstream effects that persist beyond breeding and contribute to the maintenance of pair bonds in the non-breeding season; indeed, accumulated effects of past interactions are known to be important in shaping future relationships (Hinde 1979). Testing this hypothesis, however, will require more detailed knowledge of the physiological effects of allopreening (see above).

Previous studies have suggested that allopreening may play an important role in social species (Cote and Poulin 1995; Spottiswoode 2008). In many colonially breeding species, large numbers of individuals nest in close proximity, with each pair occupying a very small breeding territory. Harrison (1965) reported that species breeding under such conditions were more likely to allopreen and argued that allopreening evolved to reduce aggression within and between breeding partners that arises as a consequence of enforced proximity (Harrison 1965). Observations of common guillemots (*Uria aalge*) provide support for this idea, where high rates of aggression among neighboring birds are associated with low rates of allopreening (Birkhead 1978; Lewis et al. 2007). In the present study, however, we found no association between allopreening and colonial breeding. The discrepancy between our results and those of Harrison (1965) is likely due to the fact that our analyses controlled for phylogeny; when phylogeny is not accounted for, we similarly find a positive association between colonial breeding and allopreening (GLM: *z* = 2.70, *P* < 0.01, *n* = 166).

An association between social breeding and allopreening has also been suggested, based on the hygienic benefits that allopreening provides (Brooke 1985; Villa et al. 2016). Focusing on cooperatively breeding species, Spottiswoode (2008) suggested that allopreening may have evolved in response to the increased risk of parasite transmission that results from close contact among group members (an argument that also holds for non-cooperative species breeding in dense colonies). However, while there is evidence that cooperatively-breeding bird species invest more in immune defenses, potentially in response to increased risk of disease transmission (Spottiswoode 2008), we found no evidence that such species are more likely to allopreen than solitary-breeding species. Though we focused on the occurrence of allopreening between breeding pairs, our literature search did not identify any species where allopreening was absent between breeders but occurred among other adult group members. Thus, the occurrence of allopreening between breeders provides an accurate guide to the presence or absence of allopreening within the group as a whole.

## CONCLUSIONS

Across bird species, allopreening is associated with parental cooperation over offspring care and partner retention across breeding attempts. The interactions that establish and maintain pair bonds in birds have previously received little attention and we hope our results will stimulate further research into the mechanisms by which allopreening influences the avian pair bond, for example through parasite removal or stress reduction. The present study focused only on the presence or absence of allopreening, but there is also likely to be variation in the amount of within-pair allopreening between species. However, data on intraspecific variation in allopreening currently exist for only a handful of species. Quantifying variation in the amount of allopreening within a greater number of species and relating this to variation in parental care and partner retention would therefore be valuable for further elucidating the adaptive significance of allopreening.

## SUPPLEMENTARY MATERIAL

Supplementary data are available at *Behavioral Ecology* online.

## FUNDING

This work was supported by a University of Sheffield Animal and Plant Sciences PhD Scholarship to E.K., and a research grant from the Natural Environment Research Council (NE/I027118/1) to J.P.G.

## Supplementary Material

Kenny_ESM_TextS1

Kenny_ESM_TableS3

Kenny_ESM_PhylogeneticTrees

Kenny_ESM_TableS2

Kenny_ESM_FigureS2

Kenny_ESM_FigureS1

Kenny_ESM_TableS1

## References

[CIT0001] Aureli F, Preston SD, de Waal FB 1999 Heart rate responses to social interactions in free-moving rhesus macaques (*Macaca mulatta*): a pilot study. J Comp Psychol. 113:59–65. 10098269 10.1037/0735-7036.113.1.59

[CIT0002] Aureli F, Yates K 2010 Distress prevention by grooming others in crested black macaques. Biol Lett. 6:27–29. 19710054 10.1098/rsbl.2009.0513 PMC2817241

[CIT0003] Birkhead TR 1978 Behavioural adaptations to high density nesting in the common guillemot *Uria aalge*. Anim Behav. 26:321–331.

[CIT0004] Black JM 1996 Partnerships in birds: the study of monogamy. Oxford (UK): Oxford University Press.

[CIT0005] Boccia ML, Reite M, Laudenslager M 1989 On the physiology of grooming in a pigtail macaque. Physiol Behav. 45:667–670. 2756061 10.1016/0031-9384(89)90089-9

[CIT0006] Boucaud ICA, Mariette MM, Villain AS 2016 Vocal negotiation over parental care? Acoustic communication at the nest predicts partners’ incubation share. Biol J Linn Soc. 117:322–336.

[CIT0007] Boyd EM 1951 The external parasites of birds: a review. Wilson Bull. 63:363–369.

[CIT0008] Brooke M de L 1985 The effect of allopreening on tick burdens of molting Eudyptid penguins. Auk 102:893–895.

[CIT0009] Carter C, Lederhendler I, Kirkpatrick B 1999 The integrative neurobiology of affiliation. Cambridge (MA): MIT Press. 10.1111/j.1749-6632.1997.tb51909.x 9071340

[CIT0010] Cornwallis CK, West SA, Davis KE, Griffin AS 2010 Promiscuity and the evolutionary transition to complex societies. Nature 466:969–972. 20725039 10.1038/nature09335

[CIT0011] Cote IM, Poulin R 1995 Parasitism and group-size in social animals: a meta-analysis. Behav Ecol. 6:159–165.

[CIT0012] Crockford C, Wittig R, Langergraber K, Ziegler T, Zuberbühler K, Deschner T 2013 Urinary oxytocin and social bonding in related and unrelated wild chimpanzees. Proc. R. Soc. B 280:20122765. 10.1098/rspb.2012.2765 PMC3574389 23345575

[CIT0013] Dagg AI 2011 Fathers and sons, and social grooming and preening. In: Animal friendships. 3rd ed. Cambidge (UK): Cambridge University Press p. 121–136.

[CIT0014] Downing PA, Cornwallis CK, Griffin AS, Griffin AS 2015 Sex, long life and the evolutionary transition to cooperative breeding in birds. Proc. R. Soc. B 282:20151663. 10.1098/rspb.2015.1663 PMC4614776 26400743

[CIT0015] Dunbar RIM 1991 Functional significance of social grooming in primates. Folia Primatol. 57:121–131.

[CIT0016] Dunbar RIM 2010 The social role of touch in humans and primates: behavioural function and neurobiological mechanisms. Neurosci. Biobehav. Rev. 34:260–268. 18662717 10.1016/j.neubiorev.2008.07.001

[CIT0017] Gill SA 2012 Strategic use of allopreening in family-living wrens. Behav Ecol Sociobiol. 66:757–763.

[CIT0018] Griggio M, Hoi H 2011 An experiment on the function of the long-term pair bond period in the socially monogamous bearded reedling. Anim Behav. 82:1329–1335.

[CIT0019] Guillerme T, Healy K 2014 mulTree: a package for running MCMCglmm analysis on multiple trees [cited 2016 March 13]. Available from: https://github.com/TGuillerme/mulTree.

[CIT0020] Hadfield JD, Nakagawa S 2010 General quantitative genetic methods for comparative biology: Phylogenies, taxonomies and multi-trait models for continuous and categorical characters. J Evol Biol. 23:494–508. 20070460 10.1111/j.1420-9101.2009.01915.x

[CIT0021] Handel CM, Gill RE 2000 Mate fidelity and breeding site tenacity in a monogamous sandpiper, the black turnstone. Anim. Behav. 60:471–481. 11032650 10.1006/anbe.2000.1505

[CIT0022] Harrison CJO 1965 Allopreening as agonistic behaviour. Behaviour 24:161–208.

[CIT0023] Harrison K, Harrison GH 1997 Birds do it, too: the amazing sex life of birds. Minocqua (WI): Willow Creek Press.

[CIT0024] Henzi SP, Barrett L 1999 The value of grooming to female primates. Primates. 40:47–59. 23179531 10.1007/BF02557701

[CIT0025] Hinde RA 1979 Towards understanding relationships. London (UK): Academic Press, in cooperation with European Association of Experimental Social Psychology.

[CIT0026] Hirschenhauser K 2012 Testosterone and partner compatibility: evidence and emerging questions. Ethology 118:799–811.

[CIT0027] Houston AI, Székely T, McNamara JM 2005 Conflict between parents over care. Trends Ecol Evol. 20:33–38. 16701338 10.1016/j.tree.2004.10.008

[CIT0028] Insel TR 2010 The challenge of translation in social neuroscience: a review of oxytocin, vasopressin, and affiliative behavior. Neuron. 65:768–779. 20346754 10.1016/j.neuron.2010.03.005 PMC2847497

[CIT0029] Jeschke JM, Kokko H 2008 Mortality and other determinants of bird divorce rate. Behav Ecol Sociobiol. 63:1–9.

[CIT0030] Jetz W, Thomas GH, Joy JB, Hartmann K, Mooers AO 2012 The global diversity of birds in space and time. Nature. 491:444–448. 23123857 10.1038/nature11631

[CIT0031] Kenny E, Birkhead TR, Green JP 2017 Data from: Allopreening in birds is associated with parental cooperation over offspring care and stable pair bonds across years. Dryad Digital Repository. http://dx.doi.org/10.5061/dryad.rn0bp. 10.1093/beheco/arx078 PMC5873249 29622926

[CIT0032] Keverne EB, Martensz ND, Tuite B 1989 Beta-endorphin concentrations in cerebrospinal fluid of monkeys are influenced by grooming relationships. Psychoneuroendocrinology. 14:155–161. 2525263 10.1016/0306-4530(89)90065-6

[CIT0033] Klatt JD, Goodson JL 2013 Oxytocin-like receptors mediate pair bonding in a socially monogamous songbird. Proc Biol Sci. 280:20122396. 23173212 10.1098/rspb.2012.2396 PMC3574448

[CIT0034] Lack DL 1940 Courtship Feeding in Birds. Auk 57:169–178.

[CIT0035] Leu ST, Burzacott D, Whiting MJ, Bull CM 2015 Mate Familiarity Affects Pairing Behaviour in a Long-Term Monogamous Lizard: Evidence from Detailed Bio-Logging and a 31-Year Field Study. Ethology 121:760–768.

[CIT0036] Lewis S, Roberts G, Harris MP, Prigmore C, Wanless S 2007 Fitness increases with partner and neighbour allopreening. Biol Lett. 3:386–389. 17550875 10.1098/rsbl.2007.0258 PMC2390679

[CIT0037] Lim MM, Young LJ 2006 Neuropeptidergic regulation of affiliative behavior and social bonding in animals. Horm Behav. 50:506–517. 16890230 10.1016/j.yhbeh.2006.06.028

[CIT0038] Mandal FB 2015 Socio-biology and social behaviour. In: Das S, editor. Textbook of animal behaviour. 3rd ed. Delhi: PHI Learning Private Limited p. 209.

[CIT0039] McFarland R, Majolo B 2013 Coping with the cold: predictors of survival in wild Barbary macaques, Macaca sylvanus. Biol. Lett. 9:20130428. 23804292 10.1098/rsbl.2013.0428 PMC3730655

[CIT0040] Moody AT, Wilhelm SI, Cameron-MacMillan ML, Walsh CJ, Storey AE 2005 Divorce in common murres (Uria aalge): Relationship to parental quality. Behav. Ecol. Sociobiol. 57:224–230.

[CIT0041] Orme D, Freckleton FP, Thomas GH, Petzoldt T, Fritz S, Isaac N, Pearse W 2013 The caper package: comparative analysis of phylogenetics and evolution in R. p. 1–36 [cited 2016 March 10]. Available from: https://cran.r-project.org/web/packages/caper/vignettes/caper.pdf.

[CIT0042] Pagel M 1994 Detecting correlated evolution on phylogenies: a general method for the comparative analysis of discrete characters. Proc R Soc B 255:37–45.

[CIT0043] Pagel M 1999 Inferring the historical patterns of biological evolution. Nature 401:877–884. 10553904 10.1038/44766

[CIT0044] Pagel M, Meade A 2006 Bayesian analysis of correlated evolution of discrete characters by reversible-jump Markov chain Monte Carlo. Am Nat. 167:808–825. 16685633 10.1086/503444

[CIT0045] R Core Team 2013 R: A language and environment for statistical computing. Vienna (Austria): R Foundation for Statistical Computing ISBN 3-900051-07-0, Available from: http://www.R-project.org/.

[CIT0046] Radford AN 2008 Duration and outcome of intergroup conflict influences intragroup affiliative behaviour. Proc Biol Sci. 275:2787–2791. 18765344 10.1098/rspb.2008.0787 PMC2605834

[CIT0047] Radford AN 2011 Preparing for battle? Potential intergroup conflict promotes current intragroup affiliation. Biol Lett. 7:26–29. 20610419 10.1098/rsbl.2010.0507 PMC3030875

[CIT0048] Remeš V, Freckleton RP, Tökölyi J, Liker A, Székely T 2015 The evolution of parental cooperation in birds. Proc Natl Acad Sci. 112:13603–13608. 26483476 10.1073/pnas.1512599112 PMC4640770

[CIT0049] Romero T, Nagasawa M, Mogi K, Hasegawa T, Kikusui T 2014 Oxytocin promotes social bonding in dogs. Proc Natl Acad Sci. 111:9085–9090. 24927552 10.1073/pnas.1322868111 PMC4078815

[CIT0050] Ross HE, Young LJ 2009 Oxytocin and the neural mechanisms regulating social cognition and affiliative behavior. Front Neuroendocrinol. 30: 534–547. 19481567 10.1016/j.yfrne.2009.05.004 PMC2748133

[CIT0051] Sánchez-Macouzet O, Rodríguez C, Drummond H 2014 Better stay together: pair bond duration increases individual fitness independent of age-related variation. Proc Biol Sci. 281:20132843. 24827435 10.1098/rspb.2013.2843 PMC4046394

[CIT0052] Sapolsky RM, Alberts SC, Altmann J 1997 Hypercortisolism associated with social subordinance or social isolation among wild baboons. Arch Gen Psychiatry. 54:1137–1143. 9400351 10.1001/archpsyc.1997.01830240097014

[CIT0053] Seyfarth RM, Cheney DL 1984 Grooming, alliances and reciprocal altruism in vervet monkeys. Nature. 308:541–543. 6709060 10.1038/308541a0

[CIT0054] Silk JB, Beehner JC, Bergman TJ, Crockford C, Engh AL, Moscovice LR, Wittig RM, Seyfarth RM, Cheney DL 2009 The benefits of social capital: close social bonds among female baboons enhance offspring survival. Proc Biol Sci. 276:3099–3104. 19515668 10.1098/rspb.2009.0681 PMC2817129

[CIT0055] Silk JB, Beehner JC, Bergman TJ, Crockford C, Engh AL, Moscovice LR, Wittig RM, Seyfarth RM, Cheney DL 2010 Strong and consistent social bonds enhance the longevity of female baboons. Curr Biol. 20:1359–1361. 20598541 10.1016/j.cub.2010.05.067

[CIT0056] Song Z, Feldman MW 2013 The coevolution of long-term pair bonds and cooperation. J Evol Biol. 26:963–970. 23496797 10.1111/jeb.12111

[CIT0057] Spoon TR, Millam JR, Owings DH 2006 The importance of mate behavioural compatibility in parenting and reproductive success by cockatiels, Nymphicus hollandicus. Anim Behav. 71:315–326.

[CIT0058] Spottiswoode CN 2008 Cooperative breeding and immunity: a comparative study of PHA response in African birds. Behav Ecol Sociobiol. 62:963–974.

[CIT0059] Tanaka I, Takefushi H 1993 Elimination of external parasites (lice) is the primary function of grooming in free-ranging Japanese macaques. Anthropol Sci. 101:187–193.

[CIT0060] Taylor SE 2006 Tend and befriend: Biobehavioral bases of affiliation under stress. Curr Dir Psychol Sci. 15:273–277.

[CIT0061] Tiddi B, Aureli F, Schino G 2012 Grooming up the hierarchy: The exchange of grooming and rank-related benefits in a new world primate. PLoS One 7:3–8. 10.1371/journal.pone.0036641 PMC3348124 22590582

[CIT0062] Villa SM, Goodman GB, Ruff JS, Clayton DH 2016 Does allopreening control avian ectoparasites? Biol Lett. 12:20160362. 27460233 10.1098/rsbl.2016.0362 PMC4971174

[CIT0063] Williams JR, Insel TR, Harbaugh CR, Carter CS 1994 Oxytocin administered centrally facilitates formation of a partner preference in female prairie voles (*Microtus ochrogaster*). J Neuroendocrinol. 6:247–250. 7920590 10.1111/j.1365-2826.1994.tb00579.x

[CIT0064] Wittig RM, Crockford C, Lehmann J, Whitten PL, Seyfarth RM, Cheney DL 2008 Focused grooming networks and stress alleviation in wild female baboons. Horm Behav. 54:170–177. 18396288 10.1016/j.yhbeh.2008.02.009 PMC4280186

[CIT0065] Xie W, Lewis PO, Fan Y, Kuo L, Chen MH 2011 Improving marginal likelihood estimation for bayesian phylogenetic model selection. Syst Biol. 60:150–160. 21187451 10.1093/sysbio/syq085 PMC3038348

