# Supplementary material for: Allopreening in birds is associated with parental cooperation over offspring care and stable pair bonds across years
Source: Behav Ecol. 2017 Jun 9;28(4):1142–8. doi: 10.1093/beheco/arx078 (PMC5873249; doi:10.1093/beheco/arx078)
Supplement: Kenny_ESM_TextS1 [file arx078_suppl_kenny_esm_texts1.docx]

**Supplementary Information to**

**Allopreening in birds is associated with cooperation over offspring care and stable bonds across years**

Elspeth Kenny^1†^, Tim R. Birkhead^1^, Jonathan P. Green^1,2^

*^1^Animal and Plant Sciences Department, University of Sheffield, Sheffield S10 2TN, UK*

*^2^ Edward Grey Institute, Department of Zoology, University of Oxford, Oxford OX1 3PS, UK*

**Text S1:**

**Supplementary Methods**

1. **Data collection**

We collated information on the following measures of pair bond strength: relative parental investment in offspring care, duration of offspring care, annual divorce rate, extra-pair paternity and pair-bond continuity throughout the year. We also collected data on breeding systems, specifically the occurrence of colonial and cooperative breeding.

- - 1. Investment in offspring care

Parental cooperation, defined here as the extent of biparental care, varies along a continuum from approximately equal investment by the male and female to obligate uniparental care (Clutton-Brock 1991). We used parental cooperation scores calculated by Remeš et al. (2015), which reflect the relative contribution by each pair member across eight activities: nest building, incubation, nest guarding, chick brooding, chick feeding, chick guarding, post-fledging feeding of chicks, and post-fledging guarding of chicks. The resulting parental cooperation score ranged from minimum parental cooperation (-1.5, where all activities are carried out by one pair member i.e. uniparental care) to maximum parental cooperation (+1.5, where all parental duties are shared approximately equally between the parents). For detail on score calculation methods see Remeš et al. (2015).

In species where both parents provide some form of care to offspring, pair bond strength may reflect not only the level of cooperation between parents but also the duration of care required to produce independent offspring. We collated information on the duration of incubation and the duration from hatching to independence (in days). Incubation period and age of independence were positively correlated (Pearson product moment correlation: r = 0.5, t = 6.81, df = 136, p<0.001), so we added these together to create a new variable, ‘total offspring care’, to encompass the total time offspring are cared for by parents.

- - 1. Divorce rate

Annual divorce rate was measured as the number of divorced pairs (cases where both partners are known to be alive but have not re-paired) divided by the total number of pairs where both partners survived from one year to the next. This definition of divorce measures the likelihood of an individual of a species choosing to repair with the same partner when the partner has survived. A previous analysis found that the primary determinant of divorce rate was mortality rate: where mortality rates are high, individuals have a low probability of encountering partners from previous breeding seasons and as a result are more likely to breed with a different partner in consecutive breeding seasons (Jeschke and Kokko 2008). To account for this, we also collected information on mortality rates for all species for which divorce rates were available. If there was more than one estimate of divorce rate for a given species, the mean of the reported rates was used.

- - 1. Sexual fidelity to social partner

Pair-bond infidelity within breeding seasons was estimated as the frequency of extra-pair paternity (EPP), measured as the percentage of broods containing extra-pair offspring. Where several estimates were available for a species, for example from different years or populations, the mean value was used.

- - 1. Time together

Species that spend all year together were classified as having ‘continuous’ partnerships, while those that come together for breeding only were classified as having ‘part-time’ partnerships.

- - 1. Colonial and cooperative breeding

To test whether allopreening is more common in species where breeding occurs in groups rather than in solitary pairs, we compared allopreening behaviour between (a) species that typically breed in large, dense colonies and those that do not, and (b) species that breed in cooperative groups of more than two individuals and those that breed in single pairs.

- - 1. Presence/absence of allopreening

For species where information on one or more aspect of pair bond strength was available, we searched for data on presence/absence of allopreening. We defined allopreening as the use of one bird’s bill to preen or groom a second individual’s feathers (Harrison 1965; Radford and Du Plessis 2006). Allopreening behaviour is therefore mechanistically distinct from other superficially similar behaviours, such as the ‘ruff-sniff’ display in crested auklets *Aethia cristatella* (Gaston and Jones (1998) and H. Douglas pers. comm.) and the ‘false preening’ behaviour of mute swans *Cygnus olor* (Boase 1959), and which were not considered in this study. Furthermore, since the focus of our study was on the relationship between allopreening and aspects of the pair bond between breeding partners, we did not consider cases of allopreening among nestlings (e.g. barn owl *Tyto alba* (Roulin et al. 2016)), between parents and nestlings (e.g. wood stork *Mycteria americana* (Clark 1980)) or between adults other than the breeding pair (e.g. green woodhoopoe *Phoeniculus purpureus* (Radford and Du Plessis 2006), guillemot *Uria aalge* (Birkhead 1978)). We did not identify any species where allopreening was absent between breeders but occurred between other adult group members. For a number of species where allopreening was reported as occurring, it was not possible to determine whether this was based on observations of breeding pairs or of other individuals, potentially outside the breeding season. For these species, we assumed that allopreening occurs between breeding partners.

We performed extensive online searches using Web of Science and Google Scholar (search terms: “*allo*preen**”, “*mutual preen**”, “*allo*groom**” and “*mutual groom**”, in combination with the species’ binomial nomenclature and common name(s)). Additionally, we also collated information from published sources held at the Alexander Library of Ornithology (Bodleian Libraries, University of Oxford, UK). Finally, where information on the presence or absence of allopreening was lacking, we contacted researchers involved in long-term, detailed behavioural studies of the species in question. Our literature search indicated that, for a given species, the presence of allopreening was more likely to be reported than its absence. We therefore assumed that allopreening did not occur if no reference was made to allopreening within otherwise detailed accounts of pair-bonding behaviour. Where there was any uncertainty about whether or not a species allopreens (for example, due to difficulties observing pair-bonding behaviour in cavity-nesting species), these species were excluded from our data set. We also excluded any species where information on the presence or absence of allopreening was only available from captive populations, as behaviours in captivity may not reflect those occurring under natural conditions (e.g. (Lambrechts et al. 1999; Garner 2005)).

Sources of all variables are available in electronic supplementary material table S1.

1. Phylogenetic relationships

To account for evolutionary non-independence, 100 phylogenetic trees were extracted randomly from the 10 000 alternative avian phylogenies from the most recent comprehensive avian phylogeny (Hackett constraint, Jetz et al. (2012)). In one case we had data on two subspecies, *Phalacrocorax atriceps melanogenis* and *P. a. bransfieldensis*, but the phylogenetic trees contained *P. atriceps* only. We assumed that the two subspecies are a sister pair, so both were added to the trees with arbitrarily short branch lengths as sister species (see PhylogeneticTrees.txt in electronic supplementary material). Rather than basing our analyses on a single phylogenetic tree and assuming this tree was known without error, we instead used a distribution of 100 trees and fitted each of our models to each of these trees using the ‘mulTree’ package (Guillerme and Healy 2014) (in R version 3.2.2, R Core Team (2013)) which carries out analyses using Markov chain Monte Carlo estimation (Hadfield and Nakagawa 2010) and summarises the resulting 100 parameter estimates. The influence of evolutionary history was established for each variable by testing for the presence of a phylogenetic signal using the ‘pgls’ function in the caper package (Orme et al. 2013) which estimated Pagel’s lambda (Pagel 1999).

1. Data analysis

We ran separate MCMC models to determine the relationship between allopreening (present/absent) and the following predictors: parental cooperation score, duration of offspring care, divorce rate (with mortality rate as a covariate), extra-pair paternity, partnership duration (continuous or part-time), colonial breeding (yes/no) and cooperative breeding (yes/no). We also ran a full model containing all predictors.

Models were run for 51000 iterations with a burn-in of 1000 iterations and a thinning interval of 50. We used a weakly informative prior with expanded parameters (V = 1, nu = 1000, alpha.mu = 0, alpha.V = 1) to improve mixing and decrease autocorrelation among iterations, and variance was fixed at one because the response variable was binary (Hadfield and Nakagawa 2010). We checked the convergence of models by visually inspecting trace plots of MCMC chains and by examining autocorrelation between posterior samples. The parameter estimates we report are the posterior mode and 95% confidence intervals (lower CI – upper CI). Parameter estimates were considered statistically significant when 95% confidence intervals did not include 0.

1. Evolutionary transition analyses

We used the BAYESTRAITS DISCRETE module with MCMC sampling (Pagel 1994) to test whether the evolution of allopreening is more likely given a low divorce rate than a high divorce rate, and equal parental investment than unequal parental investment. As BAYESTRAITS requires binary characters, we assigned species that were equal to or greater than the median level of divorce as “high divorce rate” (n=87) and those that were less as “low divorce rate”, (n=87) and likewise for parental cooperation scores (“high parental cooperation” n=209; “low parental cooperation” n=209), following Cornwallis et al. (2010) and Downing et al. (2015). To test the sensitivity of this categorisation, we repeated the analyses with species divided by 10% above and below the median: species that were equal to or greater than 10% above the median level of divorce were categorised as “high divorce rate” (n=83) and those that were less as “low divorce rate” (n=91); we then assigned species that were equal to or greater than 10% below the median as “high divorce rate” (n=90) and those that were less as “low divorce rate” (n=84); and likewise for parental cooperation scores (10% above the median: “high parental cooperation” n=139; “low parental cooperation” n=279; 10% below the median: “high parental cooperation” n=283; “low parental cooperation” n=135). Categorisation of all species for each of the models is given in electronic supplementary material table S1.

Transition rates were assessed by running a Reverse Jump model, which integrates results over a model space, weighting naturally by probabilities (Pagel and Meade 2006). Models were run for 10100000 iterations, sampling once every 1000th iteration, with a burn-in of 100000 iterations. We used hyper-priors which selected parameter values from exponential prior distributions with mean values between 1 and 100, which created a flat prior density. We checked the convergence of models by visually inspecting trace plots of MCMC chains and by examining autocorrelation between posterior samples, and took posterior distributions where the harmonic mean stabilised. We combined the posterior distribution of three independent runs from each model to ensure that transition rate estimates were stable and accurate, and accounted for phylogenetic uncertainty by including the same 100 trees used in the above analyses. We compared model support using Bayes factors estimated from a stepping stone sampling procedure (Xie et al. 2011). The marginal likelihoods of the models were calculated using a stepping stone sampler in which 100 stones were drawn from a beta distribution (with alpha = 0.4 and beta = 1). Each stone was sampled for 10000 iterations. We treated Bayes factors > 2 as evidence favouring the dependent model.

The models we report were visited in >85% of iterations in the post-burnin MCMC chain, which suggests that if these models were the best, then the parameters would by default be perfectly correlated. We therefore verified that these models were the best by comparing (1) a full unrestricted model with an exponential hyper prior, and (2) a full model with the relevant parameters restricted to zero and an exponential hyper prior. We ran these test models three times to account for variation between runs, for 1010000 iterations sampling every 1000^th^ iteration after a burn-in of 10000 iterations, and compared each model using Bayes factors calculated from the stepping stone method (Xie et al. 2011). For both models, Bayes factors were >9, demonstrating that the model reached by the Reverse Jump method was the best one.

# References:

Birkhead TR. 1978. Behavioural adaptations to high density nesting in the common guillemot Uria aalge. Anim. Behav. 26:321–331.

Boase H. 1959. Notes on the display, nesting and moult of the mute swan. Br. Birds 52:114–123.

Clark ES. 1980. The attentiveness and time budget of a pair of nesting wood storks. Proc. Colon. Waterbird Gr. 3:204–215.

Clutton-Brock TH. 1991. The evolution of parental care. Oxford: Princeton University Press.

Cornwallis CK, West SA, Davis KE, Griffin AS. 2010. Promiscuity and the evolutionary transition to complex societies. Nature 466:969–972.

Downing PA, Cornwallis CK, Griffin AS, Griffin AS. 2015. Sex , long life and the evolutionary transition to cooperative breeding in birds. Proc. R. Soc. B 282:20151663.

Garner JP. 2005. Stereotypies and other abnormal repetitive behaviors: potential impact on validity, reliability, and replicability of scientific outcomes. ILAR J. 46:106–117.

Gaston AJ, Jones IL. 1998. The Auks. Oxford: Oxford University Press.

Guillerme T, Healy K. 2014. mulTree: a package for running MCMCglmm analysis on multiple trees.

Hadfield JD, Nakagawa S. 2010. General quantitative genetic methods for comparative biology: Phylogenies, taxonomies and multi-trait models for continuous and categorical characters. J. Evol. Biol. 23:494–508.

Harrison CJO. 1965. Allopreening as agonistic behaviour. Behaviour 24:161–208.

Jeschke JM, Kokko H. 2008. Mortality and other determinants of bird divorce rate. Behav. Ecol. Sociobiol. 63:1–9.

Jetz W, Thomas GH, Joy JB, Hartmann K, Mooers AO. 2012. The global diversity of birds in space and time. Nature 491:444–448.

Lambrechts MM, Perret P, Maistre M, Blondel J. 1999. Do experiments with captive non-domesticated animals make sense without population field studies? A case study with blue tits’ breeding time. Proc. R. Soc. B Biol. Sci. 266:1311–1315.

Orme D, Freckleton FP, Thomas GH, Petzoldt T, Fritz S, Isaac N, Pearse W. 2013. The caper package: comparative analysis of phylogenetics and evolution in R. :1–36.

Pagel M. 1994. Detecting correlated evolution on phylogenies: a general method for the comparative analysis of discrete characters. Proc. R. Soc. B 255:37–45.

Pagel M. 1999. Inferring the historical patterns of biological evolution. Nature 401:877–884.

Pagel M, Meade A. 2006. Bayesian analysis of correlated evolution of discrete characters by reversible-jump Markov chain Monte Carlo. Am. Nat. 167:808–825.

R Core Team. 2013. R: A language and environment for statistical computing. R Foundation for Statistical Computing, Vienna, Austria. ISBN 3-900051-07-0, URL http://www.R-project.org/.

Radford AN, Du Plessis MA. 2006. Dual function of allopreening in the cooperatively breeding green woodhoopoe, Phoeniculus purpureus. Behav. Ecol. Sociobiol. 61:221–230.

Remeš V, Freckleton RP, Tökölyi J, Liker A, Székely T. 2015. The evolution of parental cooperation in birds. Proc. Natl. Acad. Sci. 112:13603–13608.

Roulin A, des Monstiers B, Ifrid E, Da Silva A, Genzoni E, Dreiss AN. 2016. Reciprocal preening and food sharing in colour polymorphic nestling barn owls. J. Evol. Biol. 29:380–394.

Xie W, Lewis PO, Fan Y, Kuo L, Chen MH. 2011. Improving marginal likelihood estimation for bayesian phylogenetic model selection. Syst. Biol. 60:150–160.
