# Supplementary material for: Allopreening in birds is associated with parental cooperation over offspring care and stable pair bonds across years
Source: Behav Ecol. 2017 Jun 9;28(4):1142–8. doi: 10.1093/beheco/arx078 (PMC5873249; doi:10.1093/beheco/arx078)
Supplement: Kenny_ESM_TableS3 [file arx078_suppl_kenny_esm_tables3.docx]

| Evolutionary transition | | | Evolutionary transition rates | | |
| --- | --- | --- | --- | --- | --- |
|  | From | To | 10% below median | Median | 10% above median |
| q12 | Allopreening absent  Low parental cooperation | Allopreening absent  High parental cooperation | 0.011 | 0.013 | 0.032 |
| q13 | Allopreening absent  Low parental cooperation | Allopreening present  Low parental cooperation | 0.005 | 0.0013 | 0.032 |
| q21 | Allopreening absent  High parental cooperation | Allopreening absent  Low parental cooperation | 0.011 | 0.013 | 0.080 |
| q24 | Allopreening absent  High parental cooperation | Allopreening present  High parental cooperation | 0.011 | 0.013 | 0.008 |
| q31 | Allopreening present  Low parental cooperation | Allopreening absent  Low parental cooperation | 0.011 | 0.013 | 0.090 |
| q34 | Allopreening present  Low parental cooperation | Allopreening present  High parental cooperation | 0.011 | 0.013 | 0.008 |
| q42 | Allopreening present  High parental cooperation | Allopreening absent  High parental cooperation | 0.011 | 0.013 | 0.008 |
| q43 | Allopreening present  High parental cooperation | Allopreening present  Low parental cooperation | 0.010 | 0.013 | 0.002 |

Table S3a. Estimated transition rates for coevolution of allopreening with parental cooperation over offspring care: 10% below median, median and 10% above median. For the ‘10% below median’ model, ‘high’ parental cooperation scores are equal to or greater than 10% below the median score, and ‘low’ parental cooperation scores are less than 10% below the median. For the ‘median’ model, high and low parental cooperation scores are divided at the median. For the ‘10% above median’ model, ‘high’ parental cooperation scores are equal to or greater than 10% above the median score, and ‘low’ parental cooperation scores are less than 10% above the median.

Table S3b. Estimated transition rates for coevolution of allopreening with partner retention in three different models: 10% below median, median and 10% above median. For the ‘10% below median’ model, ‘high’ divorce rates are equal to or greater than 10% below the median rate, and ‘low’ divorce rates are less than 10% below the median. For the ‘median’ model, high and low divorce rates are divided at the median. For the ‘10% above median’ model, ‘high’ divorce rates equal to or greater than 10% above the median score, and ‘low’ divorce rates are less than 10% above the median.

| Evolutionary transition | | | Evolutionary transition rates | | |
| --- | --- | --- | --- | --- | --- |
|  | From | To | 10% below median | Median | 10% above median |
| q12 | Allopreening absent  Low divorce rate | Allopreening absent  High divorce rate | 0.033 | 0.031 | 0.031 |
| q13 | Allopreening absent  Low divorce rate | Allopreening present  Low divorce rate | 0.031 | 0.031 | 0.031 |
| q21 | Allopreening absent  High divorce rate | Allopreening absent  Low divorce rate | 0.033 | 0.031 | 0.031 |
| q24 | Allopreening absent  High divorce rate | Allopreening present  High divorce rate | <0.0001 | <0.0001 | <0.0001 |
| q31 | Allopreening present  Low divorce rate | Allopreening absent  Low divorce rate | <0.0001 | <0.0001 | <0.0001 |
| q34 | Allopreening present  Low divorce rate | Allopreening present  High divorce rate | 0.033 | 0.031 | 0.031 |
| q42 | Allopreening present  High divorce rate | Allopreening absent  High divorce rate | 0.031 | 0.031 | 0.031 |
| q43 | Allopreening present  High divorce rate | Allopreening present  Low divorce rate | 0.033 | 0.031 | 0.031 |
