# Supplementary material for: Allopreening in birds is associated with parental cooperation over offspring care and stable pair bonds across years
Source: Behav Ecol. 2017 Jun 9;28(4):1142–8. doi: 10.1093/beheco/arx078 (PMC5873249; doi:10.1093/beheco/arx078)
Supplement: Kenny_ESM_TableS2 [file arx078_suppl_kenny_esm_tables2.docx]

|  | Estimate (β) | Lower CI | Upper CI |
| --- | --- | --- | --- |
| Fixed terms |  |  |  |
| Intercept | -0.29 | -9.11 | 8.39 |
| Parental cooperation | 8.73 | 2.77 | 16.89 |
| Total offspring care | -0.07 | -0.19 | -0.004 |
| Divorce | -5.31 | -15.25 | 3.25 |
| Mortality | -9.93 | -25.85 | 3.71 |
| EPP | 0.18 | 0.05 | 0.33 |
| Partnership duration | 2.30 | -2.21 | 7.19 |
| Random terms |  |  |  |
| Phylogenetic variance | 2.44 | 0.003 | 10.43 |
| Residual variance | 1.00 | 1.00 | 1.00 |

Table S2. Results from the full model testing the association between presence/absence of allopreening behaviour and measures of pair-bond strength (n = 37 species). Estimates are modal estimates from 100 models. Lower CI = lower 95% confidence interval. Upper CI = upper 95% confidence interval. Parameter estimates were considered statistically significant when 95% confidence intervals did not include 0. Residual variance was set to 1.
