# Supplementary material for: Allopreening in birds is associated with parental cooperation over offspring care and stable pair bonds across years
Source: Behav Ecol. 2017 Jun 9;28(4):1142–8. doi: 10.1093/beheco/arx078 (PMC5873249; doi:10.1093/beheco/arx078)
Supplement: Kenny_ESM_FigureS2 [file arx078_suppl_kenny_esm_figures2.docx]

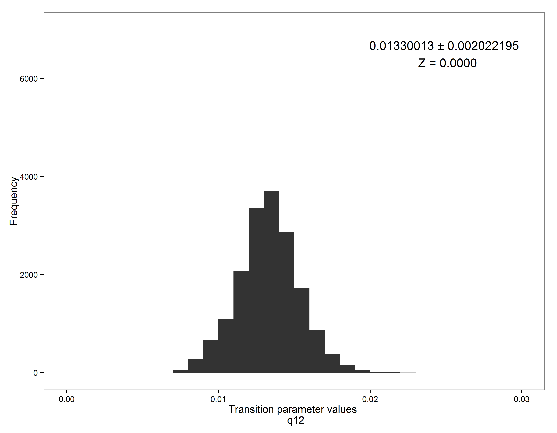

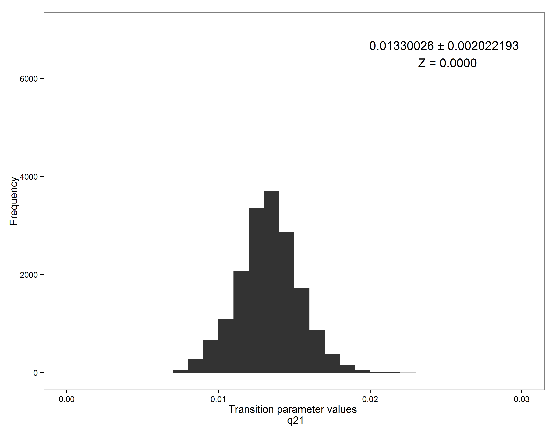

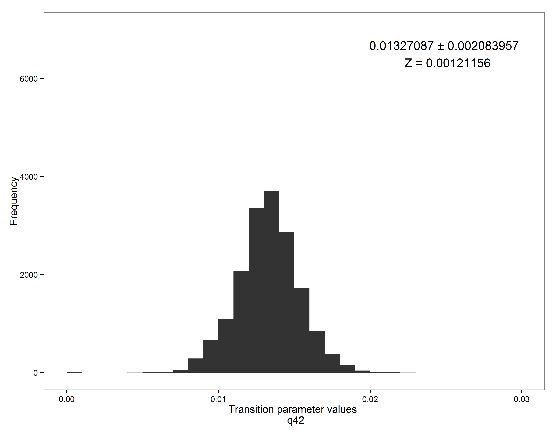

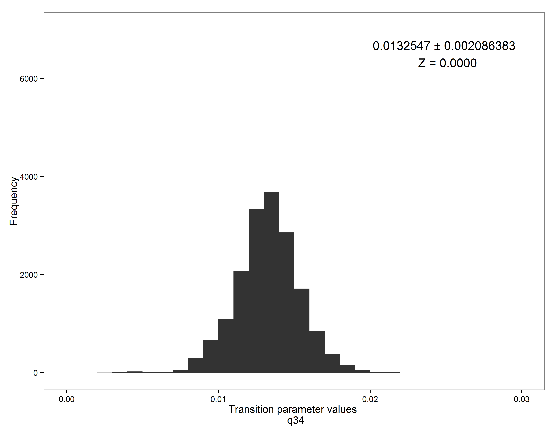

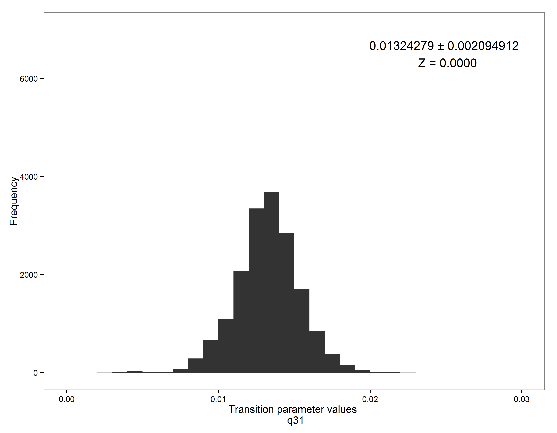

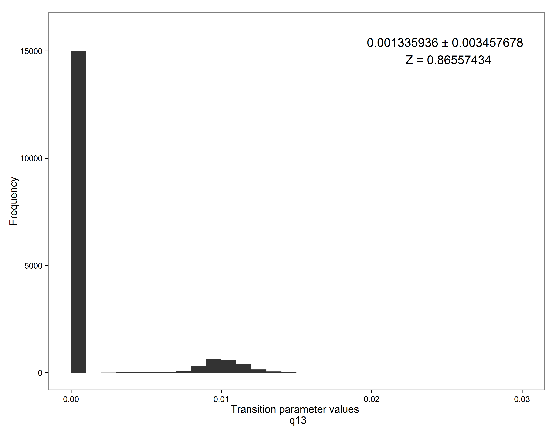

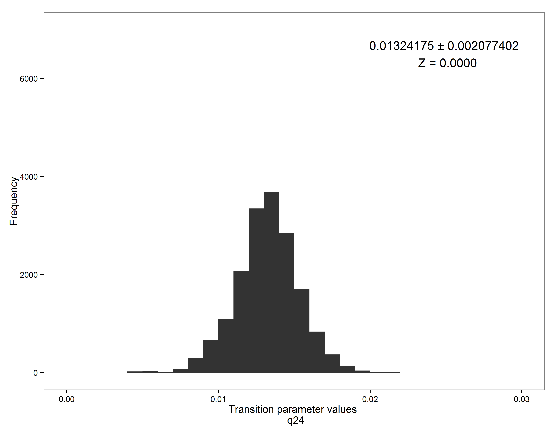

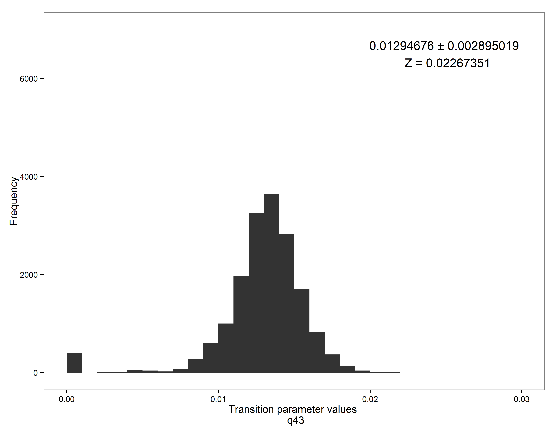


Figure S2a. Posterior probability distributions of the values of the rate coefficients of the model of correlated evolution between parental cooperation and allopreening behaviour. Z values present the proportion of the sampled runs from the Markov chain in which the parameter was assigned a value of 0. Shown are the mean and standard deviation of the parameter.


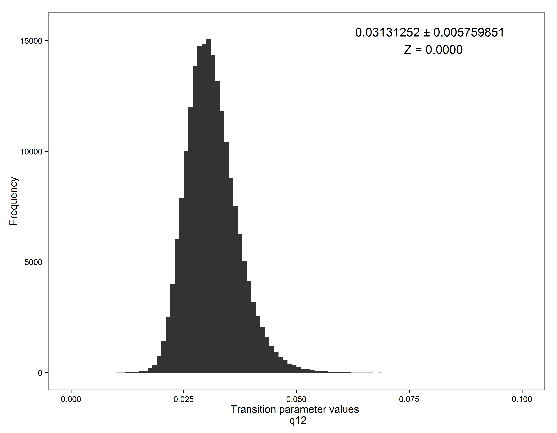

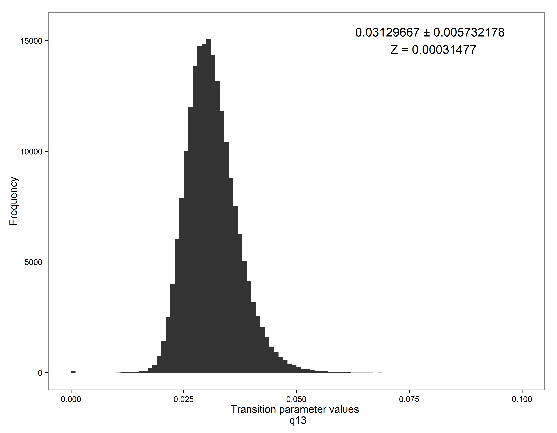

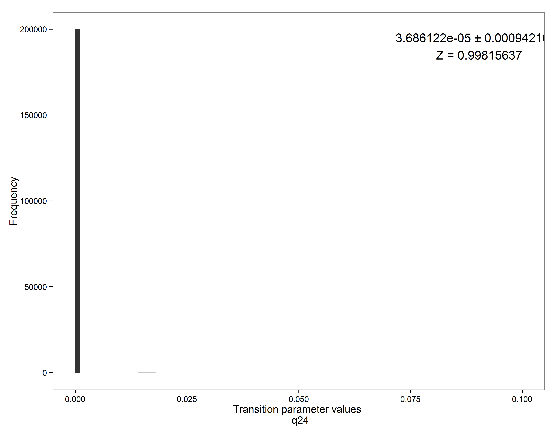

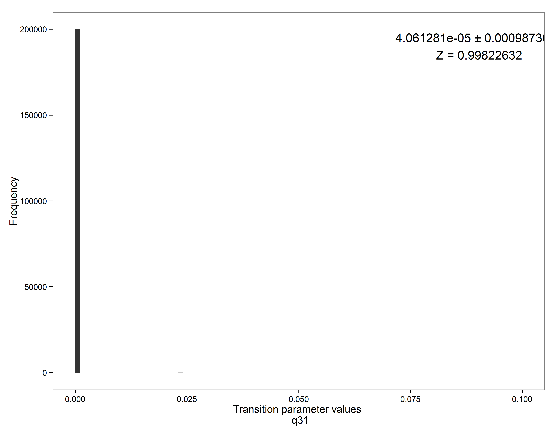

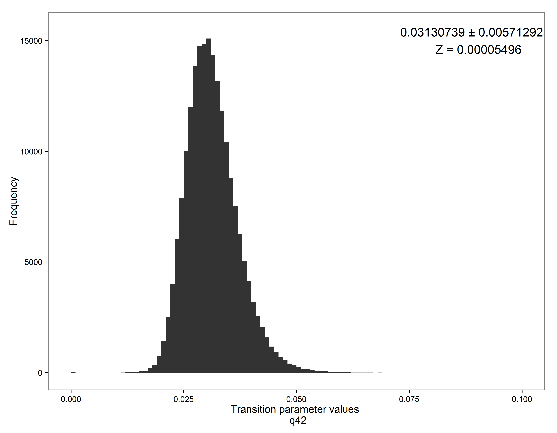

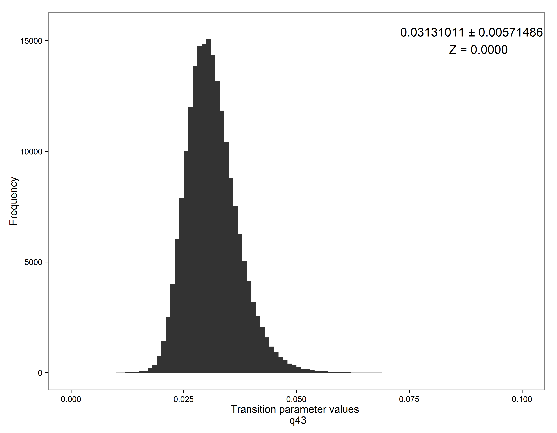

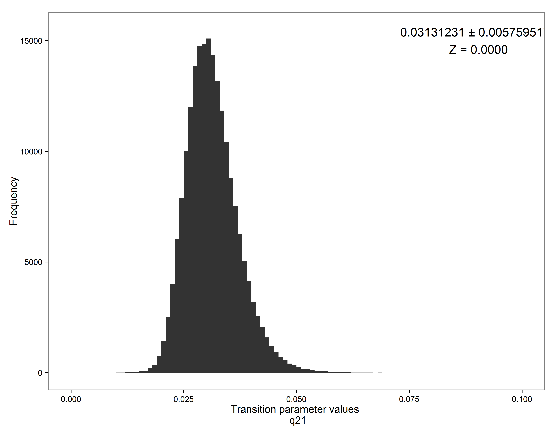

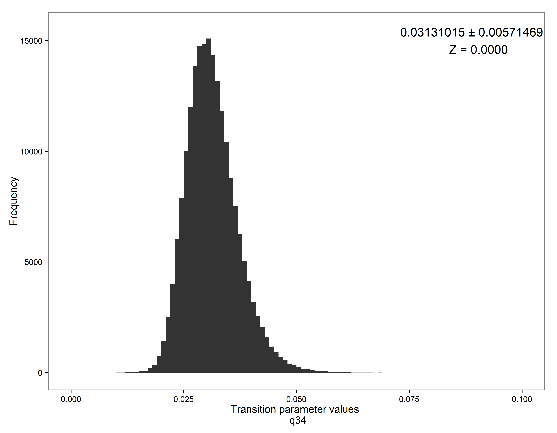


Figure S2b. Posterior probability distributions of the values of the rate coefficients of the model of correlated evolution between divorce and allopreening behaviour. Z values present the proportion of the sampled runs from the Markov chain in which the parameter was assigned a value of 0. Shown are the mean and standard deviation of the parameter.
