# Supplementary material for: Allopreening in birds is associated with parental cooperation over offspring care and stable pair bonds across years
Source: Behav Ecol. 2017 Jun 9;28(4):1142–8. doi: 10.1093/beheco/arx078 (PMC5873249; doi:10.1093/beheco/arx078)
Supplement: Kenny_ESM_FigureS1 [file arx078_suppl_kenny_esm_figures1.docx]

Allopreening absent

Low parental cooperation

(0,0)

Allopreening present

Low parental cooperation

(1,0)

Allopreening absent

High parental cooperation

(0,1)

Allopreening present

High parental cooperation

(1,1)

0.013

0.013

0.013

0.013

0.013

0.0013

0.013

0.013

a)

Allopreening absent

Low divorce rate

(0,0)

Allopreening present

Low divorce rate

(1,0)

Allopreening absent

High divorce rate

(0,1)

Allopreening present

High divorce rate

(1,1)

0.031

<0.00005

0.031

0.031

0.031

0.031

0.031

<0.00005

b)

Figure S1. Estimated transition rates for co-evolution of allopreening with (a) parental cooperation over offspring care and (b) divorce rates. ‘High’ and ‘low’ indicates whether species have divorce rates or parental cooperation scores equal to and above the median level (high) or below the median (low). Arrow thickness represents relative transition likelihood.
